# Chronic kidney disease management in patients with a failing graft: a comparative study with incident non-transplant hemodialysis patients

**DOI:** 10.1080/0886022X.2024.2447791

**Published:** 2025-01-13

**Authors:** Ana Rita Silva, Maria Guedes Marques, Luís Rodrigues, Lídia Santos, Catarina Romãozinho, Francisco Caramelo, Helena Sá, Arnaldo Figueiredo, Rui Alves, Rita Leal

**Affiliations:** a Department of Nephrology, Urology, and Kidney Transplantation, Coimbra University Hospital Center, Coimbra, Portugal; b Nephrology University Clinic, Faculty of Medicine, University of Coimbra, Coimbra, Portugal; c Biostatistics Department, Faculty of Medicine, University of Coimbra, Coimbra, Portugal; d Urology University Clinic, Faculty of Medicine, University of Coimbra, Coimbra, Portugal

Kidney allograft failure is a significant cause of end-stage kidney disease (ESKD), accounting for 5–7% of the incident dialysis patients [1, 2]. After allograft loss, patients who return to dialysis experience higher rates of morbidity and mortality compared to those who have not undergone transplantation [3, 4]. Managing a failing kidney allograft remains complex, though awareness is increasing about the importance of addressing CKD complications as the estimated glomerular filtration rate (eGFR) declines [5, 6]. Limited data comparing the management of CKD patient’s native kidney disease versus those experiencing graft failure indicate the quality-of-care metrics for kidney transplant (KT) patients with allograft loss is generally worse [7]. Our study aimed to evaluate the management of major CKD complications in the 12 months preceding hemodialysis (HD) start between two groups: those initiating HD after kidney graft loss (KT-CKD) and native kidney CKD incident HD patients (Nat-CKD).

We conducted a retrospective observational study that included all incident HD patients at a large tertiary hospital from January 2019 to December 2021. Adult patients with regular follow-up in the prior 12 months before starting HD were included. Patients were excluded if they had graft failure within the first-year post-transplant, multiorgan transplantation, or had transitioned to HD from peritoneal dialysis. We compared the rate of eGFR decline (CKD-EPI formula), and evaluated key CKD complications every three months, in the year preceding HD start. Anemia, secondary hyperparathyroidism, and nutrition parameters were registered including pharmacological management and the proportion of patients who achieved the KDIGO recommended targets [8]. We also accessed the type of vascular access and the circumstances of HD initiation: urgent dialysis, defined as immediate HD due to life-threatening conditions, and unplanned starts defined as HD initiation without an established arteriovenous fistula or graft or if HD started during hospitalization. All-cause hospitalizations (excluding surgical complications related to KT) and mortality rates were recorded in the year before and after HD initiation.

A total of 382 incident HD patients were included, 112 in the KT-CKD group and 270 in the Nat-CKD group. The KT-CKD group was significantly younger (55.9 ± 13 vs. 64.3 ± 15 years, *p* < 0.001), but there were no significant differences between groups regarding gender, race, CKD etiology, or comorbidities (Table 1). Most KT-CKD patients had received kidneys from deceased donors (92.8%) and 75.6% were on triple IS with calcineurin inhibitors, antiproliferative and steroids, before graft failure. The mean graft survival was 134 ± 93 months, with interstitial fibrosis and tubular atrophy with no specific etiology (42%) and chronic antibody mediated rejection (35%), being the most common causes of graft failure. At graft loss, all patients stopped antiproliferative drugs immediately, calcineurin inhibitors were slowly tapered and steroids were maintained for at least one-year.

**Table 1.** Demographic and clinical characteristics of patients with Nat-CKD and Tx-CKD.

	KT-CKD *N* = 112	Nat-CKD *N* = 270	P
Age (years), mean ± sd	55.9 ± 13	64.3 ± 15	<0.001
Gender (male), %	69.6%	67.8%	NS
Race (Caucasian), %	98.2%	94.4%	NS
CKD etiology			NS
Unknown	24%	18.5%	
Diabetic nephropathy	24%	29%	
ADPKD	14%	10%	
Hypertension	13%	14%	
Glomerular disease	8%	5%	
Other	17%	23.5%	
Coronary heart disease, %	36.6%	38.5%	NS
Heart failure	40.2%	42.6%	NS
Ejection fraction < 40%	7%	11%	
Diabetes	37,5%	39%	NS
Hypertension	65%	68%	NS
Neoplasia	17.9%	13.7%	NS

KT-CKD, kidney transplant chronic kidney disease; NAT-CKD, native kidney chronic kidney disease; sd, standard deviation; CKD, chronic kidney ­disease; ADPKD, autosomal dominant polycystic kidney disease.

A mixed linear model demonstrated a faster decline in eGFR among KT-CKD patients in the year before dialysis initiation, with a decline rate of 0.622 mL/min/month compared to 0.243 mL/min/month in Nat-CKD (*p* = 0.011) (Supplementary material – Figure S1). In the KT-CKD group, hypervolemia was the main reason for dialysis start (*N* = 61, 54%), while uremic symptoms were more common in the Nat-CKD group (*N* = 136, 50.5%). KT-CKD patients had significantly lower eGFR at dialysis initiation and unplanned and urgent dialysis start was more frequent. After excluding patients with a preexisting fistula or graft, the rate of KT patients with a functioning arteriovenous access at the start of HD was significant lower (Table 2). KT-CKD patients exhibited higher rates of anemia and were less likely to receive erythropoiesis-stimulating agents (ESA). Among patients with hemoglobin levels below 10 g/dL in the three months preceding HD start, only 37% of KT-CKD patients received ESA compared to 58% in the Nat-CKD group (*p* = 0.002) (Table 2). Regarding mineral bone disease, KT-CKD had higher PTH, calcium, and phosphorus levels but were less likely to receive specific treatment. Of the KT-CKD patients with PTH >300, only 30% were on vitamin D analogs or calcimimetics, compared to 70% of Nat-CKD (Table 2). Hyperphosphatemia was also less commonly managed with phosphate binders in KT-CKD patients and albumin levels were significantly lower in the KT-CKD group (Table 2). Hospitalization rates during the year prior to dialysis initiation were significantly higher in KT-CKD (49.1% vs 17.8%, *p* < 0.0001) mainly due to CKD complications (65%, *N* = 36), especially hypervolemia. The most frequent reasons for hospitalization in Nat-CKD patients were CKD complications (*N* = 17, 35%) and pre-renal acute on chronic kidney injury (*N* = 15, *N* = 31%). Post-dialysis hospitalization was also more common in KT-CKD, with KT-CKD patients having 3.2 times the risk of hospitalization compared to Nat-CKD (OR 3.2 [1.9–5.2]). In both groups, infection was the leading cause of hospitalization after starting HD. Across the entire cohort, the factors associated with hospitalization post dialysis start included previous kidney transplantation, older age, coronary artery disease, cancer, the presence of a tunneled catheter, unplanned and urgent HD start, and lower albumin level. After adjusting for these factors, multivariate analysis showed that only previous KT [OR 3.97 (IQR 2.17;7.3), *p* < 0.001] and coronary artery disease [OR 1.95 (IQR 1.1;3.4)] were independent risk factors for first-year hospitalization (Supplementary material Table S1). There was no significant difference in first-year mortality rates between the groups (19% vs 12%, *p* = 0.1), with infection and cardiovascular events being the most common causes of death in both groups (Table 2). Older age at graft loss, previous kidney transplantation, and coronary artery disease were independent risk factors for mortality.

**Table 2.** Dialysis features, chronic kidney disease complications and treatment, hospitalization, and mortality for KT-CKD and Nat-CKD.

	KT-CKD *N* = 112	Nat-CKD *N* = 270	p
eGFR at dialysis start (mL/min/1.73m^2^), mean ± sd	9,0 ± 6,0	11,1 ± 2,9	<0.001
eGFR the year before dialysis start mL/min/1.73m^2^), mean ± sd	16.6 ± 9.7	14.2 ± 5.4	<0.001
Unplanned dialysis, %, N	68% (76)	56.3% (152)	0.04
Urgent dialysis, %, N	41,1% (46)	27,8% (75)	0.015
Arteriovenous fistula/graft, %, N	46.4% (52)	52.6% (142)	0.2
New arteriovenous fistula/graft, %,N	41.5% (39)	52.6% (142)	0.04
Anemia			
Hemoglobin (g/dL), mean ± sd			
three months before	9.9 ± 0.9	10.1 ± 0.9	0.04
at dialysis start	9.6 ± 1.1	9.9 ± 0.8	0.02
Hb ≥ 10g/dL at dialysis start % (N)	27% (30)	43% (116)	0.003
ESA 3 months before dialysis % (N)	32% (36)	56% (151)	<0.001
Ferritin at dialysis start (ng/mL), mean ± sd	213 ± 97	217 ± 110	NS
CKD-MBD			
Calcium at dialysis start (mg/dL), mean ± sd	10.2 ± 0.7	9.9 ± 0.75	0.015
Phosphorus at dialysis start (mg/dL), mean ± sd	4.1 ± 0.51	3.9 ± 0.56	0.01
Treatment with phosphorus binders %, (N)	28% (31)	19% (51)	0.04
Parathormone at dialysis start (pg/mL), mean ± sd	459 ± 270	338 ± 223	<0.001
PTH > 600ng/mL (%)	17% (19)	7% (19)	<0.001
Treatment with vit D analogues (%)	41% (46)	62% (167)	<0.001
Nutrition			
Albumin at dialysis start (mg/dL); mean ± sd	3.5 ± 0.9	3.8 ± 0.6	0.003
Albumin <3.5 mg/dL (%)	27% (30)	54.5% (147)	<0.001
Hospitalization			
The year before dialysis start, % (N)	49.1% (55)	17.8% (48)	<0.0001
After dialysis start, % (N)	43% (48)	19% (51)	<0.0001
	OR 3.2 [1.9 − 5.2]		
Mortality			
First year mortality, % (N)	19% (21)	12% (33)	0.1

eGFR, estimated glomerular filtration rate; KT-CKD, kidney transplant chronic kidney disease; NAT-CKD, native kidney chronic kidney disease; sd, standard deviation; Hb, hemoglobin; ESA, erythropoiesis stimulating agents; CKD-MBD, chronic kidney disease mineral bone disorder; PTH, parathormone.

Our cohort study highlights important differences in clinical outcomes between KT-CKD and Nat-CKD with ESRDF. Despite being treated in the same clinical center, by the same nephrologist team, KT-CKD patients were less likely to receive appropriate treatment for CKD complications as their eGFR declines. We also found that a lower rate of KT-CKD patients had an arteriovenous fistula or graft at dialysis start, which resulted in higher rates of unplanned and urgent dialysis. Despite not finding an association between the worst management of CKD and the risk for hospitalization or mortality, it is important to refer that KT-CKD patients were approximately 10 years younger which should have provided a survival benefit. Also, anemia and uncontrolled hyperparathyroidism are associated with worse quality of life, and increased risk of cardiovascular events which significantly impacts patients’ outcomes in the long term. The suboptimal management of KT-CKD patients was likely due to both physician care and patient adherence. The transition from KT to dialysis disrupts many aspects of a patient’s life, affecting treatment acceptance [5]. Furthermore, transplant nephrologists are mainly focused on graft survival, but a failing graft requires a shift in care focusing on the complex transition to dialysis or re-transplantation [6].

Our study has the inherent limitations of a single center retrospective study, reflecting our specific clinical approach and the small sample size, that decreased statistical power. Also, there are important confounders when comparing two fundamentally different patient populations, including the CKD vintage and previous IS on the KT-CKD group. It should also be noticed is that the cohort was collected during the SarsCoV-2 pandemic, which had a general negative impact in the care of chronic patients.

Despite these limitations, our results reinforce the need of a shift in the paradigm of care of KT patients with a failing graft. A multidisciplinary approach is essential to address the full range of concerns when a kidney allograft is failing.

## Supplementary Material

Supplemental Material
