# Supplementary material for: Chronic kidney disease management in patients with a failing graft: a comparative study with incident non-transplant hemodialysis patients
Source: Ren Fail. 2025 Jan 13;47(1):2447791. doi: 10.1080/0886022X.2024.2447791 (PMC11731143; doi:10.1080/0886022X.2024.2447791)
Supplement: Supplemental Material [file IRNF_A_2447791_SM1758.docx]

**Supplementary Material**


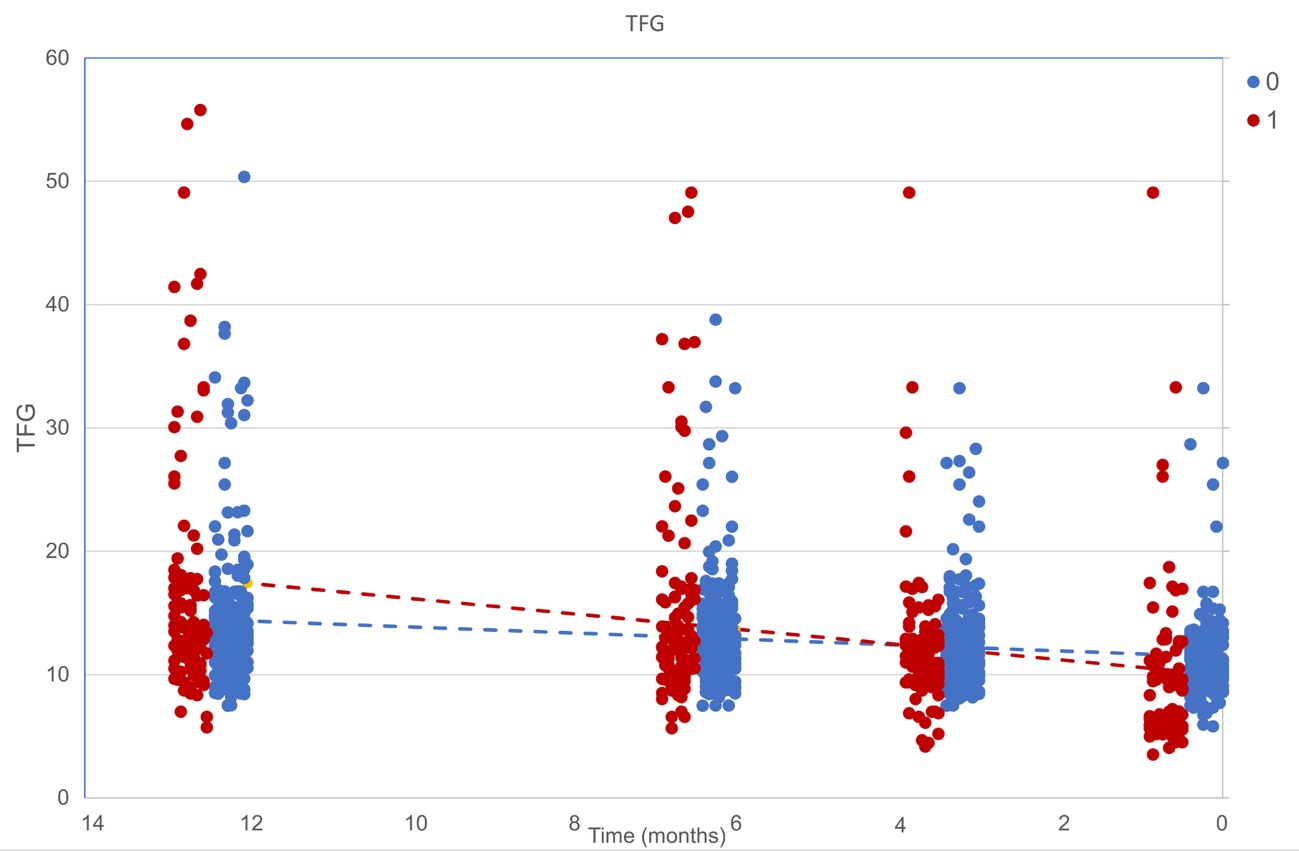


Figure S1 – eGFR distribution and decline rate the year before dialysis start for both groups. Group 0 – Native Kidney Chronic Kidney Disease (Nat-CKD); Group 1 – Kidney Transplant Chronic Kidney Disease (KT-CKD).

eGFR (mL/min/1.73m^2^)

Table S1- Risk factors for hospitalization first-year post graft failure

|  | **Hospitalization**  **N=99** | **No hospitalization**  **N=283** | **p** |
| --- | --- | --- | --- |
| **Age (mean±sd)** | **64.6±16** | **60.8±13.9** | **0.027** |
| Female (%,N) | 28.3% (28) | 32.9% (93) | 0.45 |
| **KT-CKD (%,N)** | **48.5% (48)** | **22.6% (64)** | **<0.001** |
| Multivariate analysis* | OR 3.97 [2.17;7.3] | | **<0.001** |
| **Coronary disease** (%,N) | **55.6% (55)** | **31.8% (90)** | **<0.001** |
| Multivariate analysis* | OR 1.95 (IQR 1.1;3.4) | | **<0.001** |
| **Neoplasia** (%,N) | **27.3% (27)** | **10.6% (30)** | **<0.001** |
| eGFR at T0 (mean±sd) | 10.1±4.5 | 10.6±4.1 | 0.39 |
| **Tunneled catheter** (%,N) | **64.6% (64)** | **43.8% (124)** | **<0.001** |
| **Unplanned HD** (%,N) | **74.7% (74)** | **54.4% (154)** | **<0.001** |
| **Urgent HD** (%,N) | **45.5% (45)** | **27% (76)** | **<0.001** |
| **Albumin at T0 (mean±sd)** | **3.5±0.6** | **3.75±0.69** | **0.008** |
| **Albumin <3.5** (%,N) | **42.4% (57)** | **32.5%** | **0.05** |
| **Hemoglobin ≥10g/dL** (%,N) | **33.3% (33)** | **40.3% (114)** | **0.135** |
| Ferritin at T0 (mean±sd) | 219±115 | 215±103 | 0.721 |
| Calcium 8.5-10.5mg/dL (%,N) | 73.7% (73) | 72.4% (205) | 0.89 |
| Phosphorus at T0 (mean±sd) | 3.95±0.63 | 3,97±0.52 | 0.74 |
| PTH<300ng/mL (mean±sd | 34.7% (34) | 42.4% (129) | 0.111 |

Sd-standard deviation; KT-CKD – kidney transplant patients returning to hemodialysis; eGFR – estimated glomerular filtration rate; HD-hemodialysis; PTH – parathormone.

Multivariate analysis was performed using a model that included all variables with differences p<0.1 between groups. The model was statistically significant.
